# [Retracted] Morphine‑induced delayed pre‑conditioning against anoxia/reoxygenation injury in pulmonary artery endothelial cells: The role of mitochondrial KATP channels

**DOI:** 10.3892/mmr.2023.13030

**Published:** 2023-06-12

**Authors:** Wengang Ding, Yueping Guo, Xiaoguang Cui, Bing Zhang, Dongmei Li, Wenzhi Li

Mol Med Rep 13: 1047–1053, 2016; DOI: 10.3892/mmr.2015.4629

Following the publication of this paper, it was drawn to the Editor's attention by a concerned reader that several of the flow cytometric plots featured in Fig. 2A on p. 1050 contained repeating patterns of dots, both vertically and horizontally, in addition to a variety of other apparent anomalies.

The authors were asked to provide an explanation to account for the apparent anomalies in this figure, but they did not respond to the request posed by the Editorial Office. Therefore, the Editor of *Molecular Medicine Reports* has decided that this paper should be retracted from the journal on account of a lack of confidence in the presented data. The Editor apologizes to the readership for any inconvenience caused.

